# Supplementary material for: Metatranscriptomic Analysis Reveals Rich Mycoviral Diversity in Three Major Fungal Pathogens of Rice
Source: Int J Mol Sci. 2022 Aug 16;23(16):9192. doi: 10.3390/ijms23169192 (PMC9409214; doi:10.3390/ijms23169192)
Supplement: Supplementary file 1 [file ijms-23-09192-s001.zip › Table S1.pdf]

Table S1. Statistics of assembly sequences from metatranscriptomic sequencing data of three major fungal pathogens of rice.

Sample	Raw reads	Clean reads	Total contigs	Viral-related contigs
M. oryzae	2.16×10^8	2.11×10^8	116,736	79
U. virens	2.88×10^8	2.84×10^8	106,131	593
R. solani	7.82×10^7	7.70×10^7	99,590	10
